# Visualization of Sugar Content Distribution of White Strawberry by Near-Infrared Hyperspectral Imaging

**DOI:** 10.3390/foods12050931

**Published:** 2023-02-22

**Authors:** Hayato Seki, Te Ma, Haruko Murakami, Satoru Tsuchikawa, Tetsuya Inagaki

**Affiliations:** 1 Institute of Agricultural Machinery, National Agricultural and Food Research Organization, 1-40-2, Nisshin-Cho, Kita-Ku, Saitama City 331-8537, Japan; 2 Graduate School of Bioagricultural Sciences, Nagoya University, Furo-Cho, Chikusa, Nagoya 464-8601, Japan

**Keywords:** white strawberry, hyperspectral imaging, principal component analysis, image processing, partial least squares regression, sugar content distribution

## Abstract

In this study, an approach to visualize the spatial distribution of sugar content in white strawberry fruit flesh using near-infrared hyperspectral imaging (NIR-HSI; 913–2166 nm) is developed. NIR-HSI data collected from 180 samples of “Tochigi iW1 go” white strawberries are investigated. In order to recognize the pixels corresponding to the flesh and achene on the surface of the strawberries, principal component analysis (PCA) and image processing are conducted after smoothing and standard normal variate (SNV) pretreatment of the data. Explanatory partial least squares regression (PLSR) analysis is performed to develop an appropriate model to predict Brix reference values. The PLSR model constructed from the raw spectra extracted from the flesh region of interest yields high prediction accuracy with an RMSEP and $R^{2}{}_{p}$ values of 0.576 and 0.841, respectively, and with a relatively low number of PLS factors. The Brix heatmap images and violin plots for each sample exhibit characteristics feature of sugar content distribution in the flesh of the strawberries. These findings offer insights into the feasibility of designing a noncontact system to monitor the quality of white strawberries.

## 1. Introduction

Strawberries (*Fragaria × ananassa*) are a common fruit produced and consumed worldwide. Although the most apparent feature of strawberries is their red skin, strawberries with white skin (white strawberries) have recently been introduced in the Japanese market. The accumulation of Anthocyanins (pelargonidin 3-glucoside, pelargonidin 3-rutinoside, and cyanidin 3-glucoside), which are the typical red pigments in strawberries [1], is suppressed in white strawberries [2,3,4,5]. In Japan, the price and quality of strawberries are evaluated using standard criteria based on color, size, shape, damage, and taste. Their quality is generally evaluated manually, which is often inaccurate as quality validation tends to differ from person to person [6]. Therefore, with the recent concerns regarding overall food quality (such as taste, appearance, and freshness) and safety based on national and international standards, the development of automatic technologies to determine the quality of fresh strawberries has been considered [7]. Although color (i.e., redness) is an important evaluation criterion used to determine the ripeness of red strawberries, evaluating the ripeness of white strawberries from a visual inspection is challenging [5] because their color does not vary with ripeness. An increase in sugar content and a decrease in acid content occur with increasing ripeness in strawberries [8]. Strawberries’ organoleptic quality has been shown to be affected by the sensory attributes “sweetness” and “aroma” [9]. Sugar and sweetness can be determined using sensory evaluation, hydrometers, refractometers, high-pressure liquid chromatography (HPLC), electronic tongues, colorimetric methods, and other methods [10]. However, because these methods are destructive, a technique to evaluate the quality of white strawberries without destroying the sample must be developed.

Near-infrared (NIR) spectroscopy (780–2500 nm) combined with multivariate mathematical methods is effective for assessing the external quality of fruits [11]. NIR spectroscopy has also been applied to assess the internal quality of strawberries [12,13]. NIR spectroscopy is quick and simple for collecting spectral information of fruits; however, it offers only averaged spectra of target samples without providing any information about the spatial distribution. Hyperspectral imaging (HSI) generates 3D datasets of spatial and spectral data called hypercubes by collecting the spectral information of each pixel of a two-dimensional (2D) data array. These hypercubes include one spectral and two spatial dimensions [14]. By combining image processing and chemometric methods for spectroscopy, studies have successfully evaluated the distribution of solid soluble content (SSC) in apples [15,16] and bananas [17], the SSC and pH of cherries [18], the SSC and hardness of melons [19,20], and the SSC, hardness, and pH of kiwifruits [21]. HSI has been employed to determine the external quality and safe storage time of fruit by measuring ripeness, bruises and fungal infections in terms of external quality and storage time, as well as to anthocyanins, vitamin C, SSC, and pH as internal quality in red strawberries [7,22,23,24,25,26,27]. However, to the best of our knowledge, no prior works have reported using HSI to predict the quality of white strawberries. In this study, HSI in the range of short-wave infrared (SWIR) wavelengths (900–2500 nm) was used because molecular vibration information is needed to evaluate white strawberries [28]. The quality of red strawberries can be evaluated by HSI in the range of visible–NIR (Vis–NIR) wavelengths (400–1000 nm) because absorbance in the range of 400–700 nm (color information) is important for evaluation. The region-of-interest (ROI) used in these studies include pixels corresponding to the flesh (in terms of plant morphology, the accessory fruit, which has an enlarged receptacle) and achene (similar to seeds on the surface, in plant morphology, this is called a true fruit). Major soluble sugars in strawberries, including sucrose, glucose, and fructose, contain in the receptacle. However, these sugars do not contain much in the development of achenes [29]. However, in earlier works on the visualization of strawberry quality using HSI [26,29], the average spectrum calculated from the spectra of all pixels on the fruit surface, including the flesh and achene, was employed to construct regression or discriminant models, and the spectrum of each pixel was used to predict the chemical, physical, or category information of a pixel using the developed model. In this present study, first, a method to automatically classify flesh and achene based on the first principal component (PC1) score from the principal component analysis of the surface data of each fruit was developed. Image masks showing pixels of the flesh and the achene were created, the spectra of the whole fruit, flesh, and achene were extracted, and the average spectrum of each was calculated.

In this study, a combination of image processing and chemometric methods for spectra was designed for noncontact Brix evaluation in white strawberries using the NIR hyperspectral imaging system. To achieve these goals, (1) a practical and effective method that automatically extracts information on pixels showing the flesh of fruit samples by principal component analysis (PCA) imaging and binarization after appropriate preprocessing was developed; this method does not require any training data and, therefore, does not require the cost of creating correct label images; (2) a regression model was developed to forecast the degrees Brix of the flesh of fruit using partial least squares regression (PLSR); (3) the developed model for estimating the sugar content of strawberry flesh was applied to pixels showing flesh on the surface of strawberry fruit, and the estimated sugar content for each pixel was obtained. The proposed evaluation method for white strawberries includes a heatmap of the sugar content estimated for each pixel and a violin plot indicating the sugar content distribution for the whole flesh, as well as displaying the top and bottom of the flesh.

## 2. Materials and Methods

### 2.1. White Strawberry Samples

Strawberry samples of the cultivars “Tochigi iW1 go” with white skin were obtained from the Strawberry Research Institute–Tochigi Prefectural Agricultural Experiment Station (Tochigi-Shi, Tochigi Pref. 328-0007, Japan) between February and March 2021. The ripeness and shape varied among the 180 strawberries. Before the experiment, the strawberries were kept under controlled conditions at 23 °C to reduce variations in measurement caused by temperature changes. Samples were transported by refrigerated shipping after harvest and stored in a standard refrigerator for approximately 1 h before measurement. As per estimates, 1–2 d had elapsed between harvest and measurement. No serious deterioration in quality was observed visually during the experiment.

### 2.2. NIR Hyperspectral Images and Brix Measurements

**Figure 1:**
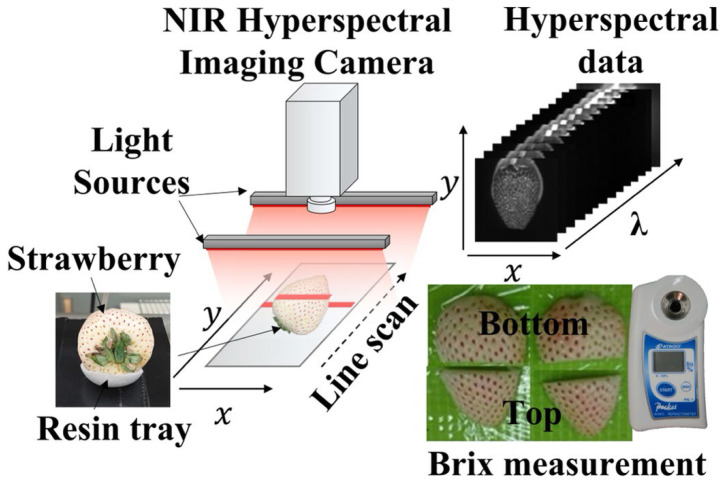
Near-infrared hyperspectral imaging (NIR-HSI) system and Brix measurement method used in this work.

Figure 1 shows an overview of the NIR hyperspectral imaging measurement (push-broom line scanning system: Compovision, Sumitomo Electric Industries, Ltd., Tokyo, Japan) and the Brix measurement methods employed in this study.

At a spectral interval of 6.2 nm, the camera was equipped with a spectroscope and a 2D photosensitive element (256 pixels (wavelength) × 320 pixels (position)) capable of receiving NIR light from 913 to 2519 nm. A wavelength ranging from 913 to 2166 nm (i.e., 200 wavelength bands) was selected herein because reflectance over 2166 nm has a low signal-to-noise (S/N) ratio. In order to attain a horizontal field of view of 50 mm for the strawberry samples, the distance between the target and the camera was adjusted with a spatial resolution of 156 μm/pixel. The light source was tube-shaped and illuminated from both sides using four halogen lamps. The irradiation angle was adjusted to 45°. Each sample was placed on a slider and scanned linewise. The frame rate was set to 30 frames s^–1^. Both sides were measured by flipping each sample 180°. A soft resin tray was placed between the slider and the sample to hold the sample in place. As a reference, a white plate was measured at 200 frames s^−1^, and dark images were measured by turning off the light source and covering the lens with a cap. The collected spectral images were converted to relative reflectance values for further analysis using Equation (1), as given below.

(1) $$R_{\lambda ,n}=\left(S_{\lambda ,n}-D_{\lambda ,n}\right)/\left(W_{\lambda ,n}-D_{\lambda ,n}\right)$$

where *λ* and *n* represent the wavelength and pixel index variables, respectively; $R_{\lambda ,n}$ represents the standardized reflectance intensity at wavelength *λ* and pixel *n*; *S* and *W* represent sample and white reference images, respectively; and D represents dark images. After measuring the hyperspectral data, each measurement surface was divided into two areas indicating the apex and base of the fruit. The fruit sections were then wrapped in a nonwoven cloth, squeezed by hand, and pressed. The juice was stirred well, and the Brix value was measured using a Brix meter (PAL-1, ATAGO Co., Ltd., Tokyo, Japan).

### 2.3. Preprocessing of Hyperspectral Images

The ROI should be predetermined to extract spectral information of strawberries from hyperspectral images. In this study, the ROI of the whole fruit, flesh, and achene in strawberries was determined. The ROI for each part was determined based on PCA and image processing.

#### 2.3.1. Creating a Fruit Mask Using Thresholding

**Figure 2:**
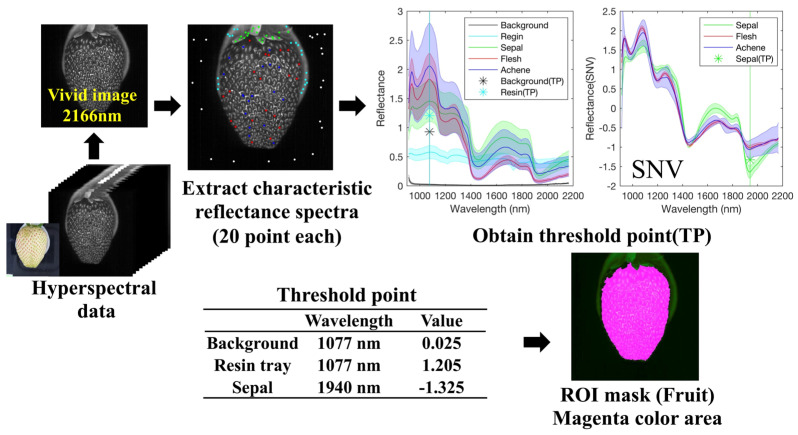
Preprocessing procedure for hyperspectral data with ROI extraction by thresholding (Fruit mask).

Figure 2 shows the method used to determine the ROI of the fruit. First, the pixels corresponding to the background, resin tray, and sepals were determined based on the reflectance value at a specific wavelength using thresholding, as shown in Figure 2. To determine the wavelength and threshold value for the recognition of the background, resin tray, and sepals, 20 pixels corresponding to the background, resin tray, sepals, and flesh achene, were manually selected, and the average and standard deviation spectra of these 20 pixels were calculated, as shown in Figure 2 (right top).

As the reflectance values at 1077 nm for sepals, flesh, and achene differed significantly from those of the resin tray and background, the reflectance value at 1077 nm was used for the separation of the resin tray and background with a threshold value of 1.205, which is the midpoint of the resin tray and flesh at 1077 nm. After removing the pixels corresponding to the resin tray and background, smoothing with the Savitzky–Golay filter (window size, 7) and standard normal variation (SNV) were conducted for the spectra at each pixel. SNV spectral preprocessing was performed on each pixel to eliminate the physical light-scattering effects and increase the spectral information [30].

(2) $$x_{i,snv}=\left(x_{i}-\bar{x}\right)/\left(\sqrt{\sum_{i=1}^{n}\left(x_{i}-\bar{x}\right)/\left(n-1\right)}\right)$$

where $x_{i,snv}$ denotes the NIR spectrum matrix after SNV pretreatment for the original spectrum $x_{i}$, and $\bar{x}$ represents the mean intensity of all wavelengths of the same spectrum. After SNV pretreatment, a significant difference was observed between the sepal part and the flesh or achene part at 1940 nm. Thus, the reflectance value after SNV at 1940 nm was used to determine the pixel corresponding to the sepal with a threshold value of −1.325, which was chosen as the midpoint value of the spectra of the sepal and flesh. Pixels with reflectance greater than 1.205 at 1077 nm and greater than −1.325 after pretreatment at 1940 nm were designated as fruit ROI (including flesh and achene).

#### 2.3.2. Determination of ROI Corresponding to Flesh Part and Achene Part Using a Combination of PCA and Image Processing

**Figure 3:**
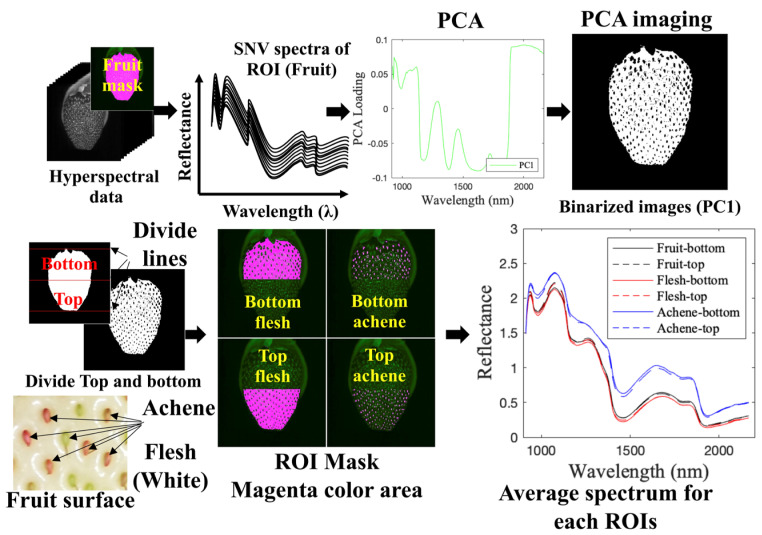
Preprocessing procedures for hyperspectral data, extraction of ROIs by PCA imaging and image processing, average spectra extracted from 6 ROIs (Fruit-bottom, Fruit-top, Flesh-bottom, Flesh-top, Achene-bottom and Achene-top).

Figure 3 depicts the proposed imaging procedure, which combines PCA and image processing to classify pixels corresponding to the flesh parts of strawberries and achenes. This process yielded the ROI corresponding to flesh and achene for the top and bottom of the fruit, which allowed us to calculate the average spectrum from the flesh and achene parts.

The raw spectra of the fruit surface were extracted using an ROI mask of only the fruit surface created by a thresholding process. PC1 loading was obtained by PCA for the spectra after smoothing using a Savitzky–Golay filter and SNV treatment. Autoscaling was performed prior to the PCA. PC1 loading was applied to the hyperspectral data to produce a PC1 image. ROI masks were determined to classify flesh and achene pixels for each sample from the PC1 image binarized using Otsu’s method [31]. Moreover, image processing was employed to determine the midpoint coordinates for dividing the fruit into the top and bottom of the fruit mask. Finally, six ROI masks (Fruit-bottom, Fruit-top, Flesh-bottom, Flesh-top, Achene-bottom, and Achene-top) were constructed from each sample, and the average spectrum of each region was computed.

### 2.4. PLSR Modeling

The dataset consisting of spectra and sugar content for each ROI (Fruit, Flesh, achene) included 720 data samples. The training and testing sets had a 1:1 ratio because one side of each sample was chosen. A total of 360 data points were used for training and 360 for prediction. PLSR was performed to develop a calibration model between the averaged NIR spectral data and the Brix reference values in the training dataset. The number of PLS factors (LVs) was determined using the 10-holdout cross-validation (CV) method. The optimal LVs were selected in terms of the maximum root-mean-square error (RMSE) for cross-validation (RMSECV) within the range of the global minimum + 1 standard deviation. The upper limit of LVs was set at 20. Moreover, the competitive adaptive reweighted sampling (CARS) method was employed to select the critical wavelengths [16] and improve the robustness of the model by reducing the number of variables. In the CARS program [32], the regression coefficients of the PLSR model were employed as an index to evaluate the contribution of each wavelength in the Brix prediction model. CARS was used to sequentially select N subsets of wavelengths from N sampling runs. In each sampling run, the number of wavelengths to be selected by CARS was regulated by the proposed exponentially decreasing function and by adaptive reweighted sampling. Finally, CARS was used to discover a combination of wavelengths with the lowest RMSECV. The model constructed for the training dataset was applied to the testing dataset to confirm the effectiveness of the model.

The quality of the PLSR model was assessed using the determination coefficient ($R^{2}$) and RMSE for calibration ($R^{2}c\;\mathrm{and}\;RMSEC$) and prediction ($R^{2}p\;\mathrm{and}\;RMSEP$). A good model possesses a low RMSEC, RMSEP, and high determination coefficient ($R^{2}{}_{c},\;R^{2}{}_{p})$ such that calibration and confirmation results do not diverge. The criteria are defined as follows.

(3) $$RMSECV,RMSEC,RMSEP=\sqrt{\left(1/n\right)\sum_{i=1}^{n}{\left({\hat{y}}_{i}-\bar{y}\right)}^{2}}$$

(4) $$R^{2}{}_{c},R^{2}{}_{p}=1-\left\{\left(\sum_{i=1}^{n}{\left(y_{i}-\hat{y}\right)}^{2}\right)/\left(\sum_{i=1}^{n}{\left(y_{i}-\bar{y}\right)}^{2}\right)\right\}$$

where *n* represents the number of samples, *y* represents the Brix values measured using the Brix meter; $\bar{y}$ denotes the mean values of *y*; and $\hat{y}$ denotes the Brix value forecast using NIR spectroscopy during calibration or confirmation. Herein, 24 patterns of model searches were used to determine the best model; these patterns (2 × 2 × 2 × 3) included SNV processing or raw, second derivative processing using the Savitzky–Golay filter or no such processing, variable selection with CARS or no such selection when the latent variables were determined with cross-validation, and three patterns of ROI (fruit, flesh, or achene). The average spectra were preprocessed using autoscaling prior to PLSR.

### 2.5. Visualization of the Sugar Content Distribution

**Figure 4:**
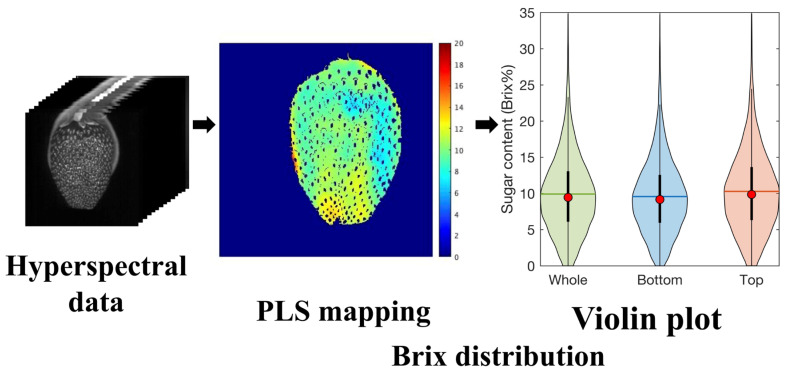
Procedure for visualization of sugar content distribution. Violin plot (colored areas: distribution of data from kernel density estimation; red dots: median; black thick vertical line: interquartile range; horizontal colored lines: mean).

The pixels corresponding to the flesh ROI in the hyperspectral images of the test data were used for sugar content visualization by applying the PLSR model constructed to estimate the Brix values. The spectra in the ROI were preprocessed by autoscaling before fitting the model, following the same procedure as that used for constructing the model. After smoothing the image using a Gaussian filter to eliminate noise, the sugar content distribution was displayed on a heat map. Moreover, in the violin plot, the distribution of sugar content in the entire strawberry flesh section, bottom of the flesh, and top of the flesh could be determined from the distribution of data based on kernel density estimation. In addition, the mean, median, and interquartile range played a role in assisting in the interpretation of this sugar distribution. Figure 4 depicts the procedure performed to visualize the sugar content distribution.

All data analyses were conducted using MATLAB 2021, a computer analysis software package [33]. CARS and PLSR were conducted using libPLSR_1.98 [32], and a violin plot was constructed using Violinplot-Matlab [34].

## 3. Results and Discussion

### 3.1. Preprocessing of Hyperspectral Images

**Figure 5:**
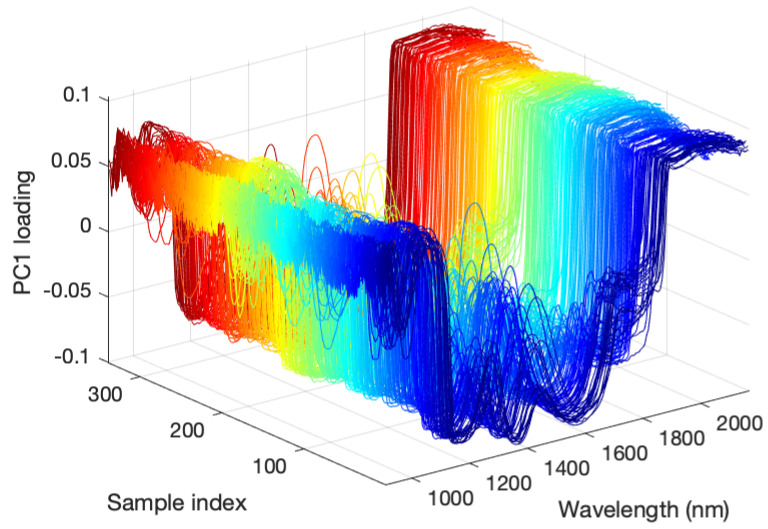
PC1 loadings of all samples obtained by principal component analysis from the pixels of the hyperspectral data measurement plane.

Figure 5 depicts PC1 loading for each sample. The PC1 loading of each sample exhibited a similar shape. In Otsu’s binarization method, the threshold value that maximizes the variance between the two classes is determined and classified into two groups. This pretreatment with PC1 loading generally distinguished the flesh and achene from the fruit into two groups. This preprocessing method is considered practical because it is an automatic discrimination method that does not require a training data set and can be performed on each strawberry surface.

**Figure 6:**
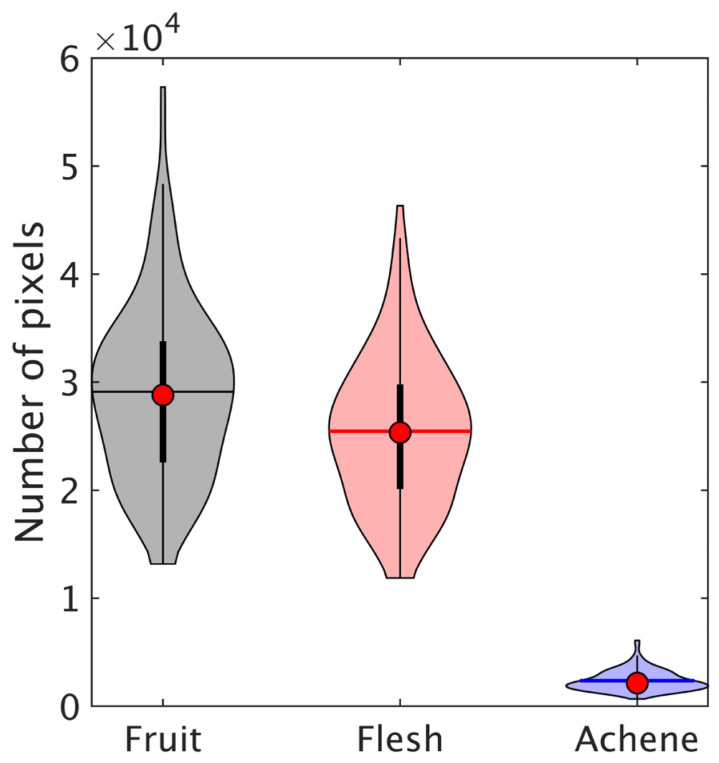
Number of pixels for ROI of fruit, flesh, and achene in strawberry, with ROIs defined by image masks created by preprocessing hyperspectral data.

Some samples included pixels where the reflectance was saturated owing to the Fresnel reflection of irradiated light on the unevenness of the strawberry surface. These pixels were eliminated and not used for further calculations. The mean values of the number of pixels assigned to the fruit, flesh, and achene parts were 29,038, 25,454, and 2367, respectively, as shown in Figure 6. The average ratio of the pixels corresponding to achenes in fruits was 8.7%. In addition, the distribution of the number of pixels was wide owing to variations in size and shape.

**Figure 7:**
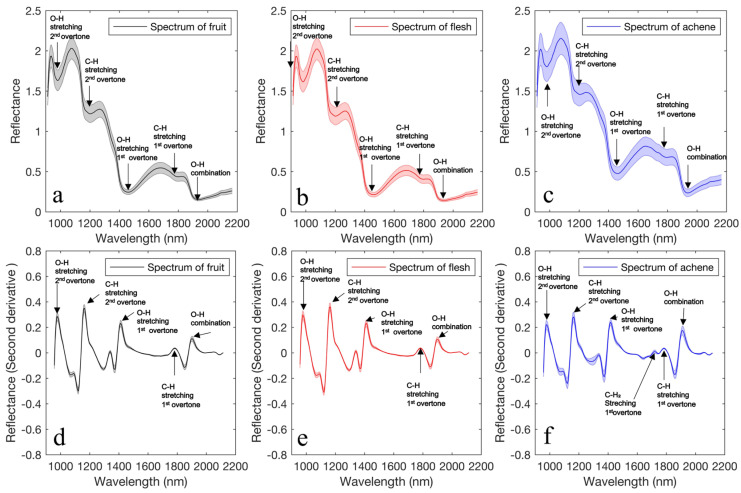
Average reflectance spectra (the spectral range is the mean ± standard deviation) of: (**a**) fruit, (**b**) flesh, and (**c**) achene. Second derivative average reflectance spectra (Spectral range is mean ± standard deviation) of: (**d**) fruit, (**e**) flesh, and (**f**) achene in strawberries.

Figure 7 shows the average spectra of: (a) fruit, (b) flesh, and (c) and achene; and their corresponding second derivative spectra ((d), (e), (f), respectively). The average spectrum had absorption peaks at 970, 1165, 1420, 1780, and 1900 nm. The peaks at 970, 1420, and 1900 nm corresponded to O–H-related water content, those at 1165 and 1780 nm corresponded to C–H, and those at 1165 and 1780 nm corresponded to C–H-related sugar [28]. These absorption bands have also been observed in red strawberries [35]. The average spectra from the flesh part exhibited different characteristics from the achene part, i.e., the reflection at 1420 nm due to water because the water content value significantly differed between flesh and achene. The fruit and flesh spectra exhibited almost identical peak intensities because pixels of achene had a low ratio to those of the fruit, at 8.7%. Flesh and achene exhibited differences in absorption peak intensity in the second derivative spectra, particularly at 1165 nm owing to CH and 970 and 1900 nm owing to OH. Furthermore, a specific absorption peak was observed only from achene at approximately 1710 nm, which corresponds to C–H_2_ [28].

### 3.2. PLSR Model

**Figure 8:**
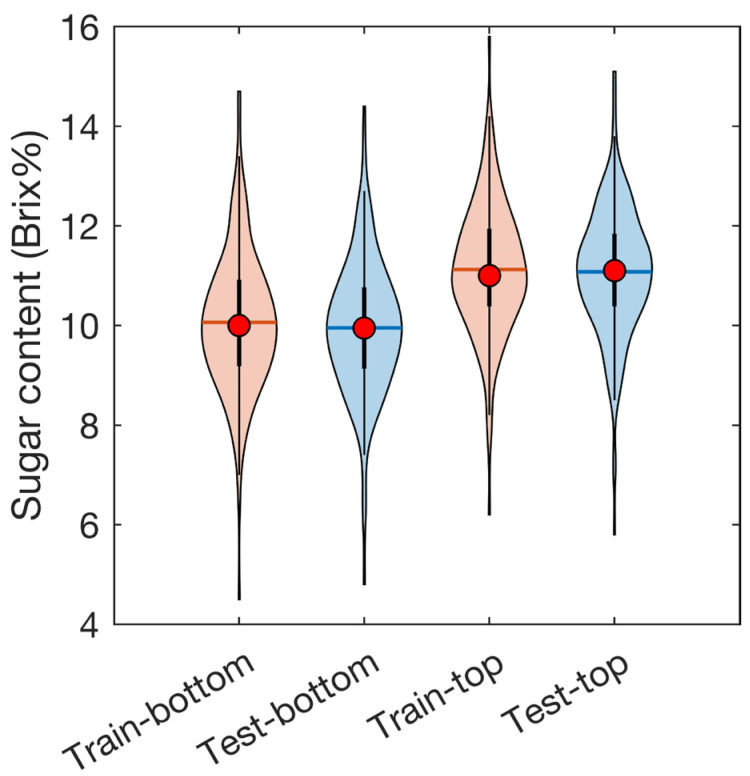
Sugar content (Brix%) references measured from blocks cut from the top and bottom of the fruit using a Brix meter (training dataset vs. testing dataset).

Figure 8 shows the distribution of Brix reference values for strawberries in the training and testing datasets from the bottom and top parts of the strawberries. The training dataset contained a more comprehensive range of values compared with the testing dataset. The Brix value at the top of the fruit was higher than that at the bottom. This result indicates that white strawberries accumulate more sugar at the top of the fruit, as do red strawberries [35].

Table 1 summarizes the PLSR model evaluated using numerous conditions (such as spectral pretreatment and ROI used). Evidently, the model constructed from the spectra extracted from the achene ROI yielded a low value of $R^{2}{}_{p}$. We considered that the relationship between the information on achene and the information on fruit sugar accumulation was not good. Based on $R^{2}{}_{p}$, the model constructed from spectra extracted from the fruit or flesh ROI exhibited a higher prediction accuracy.

**Figure 9:**
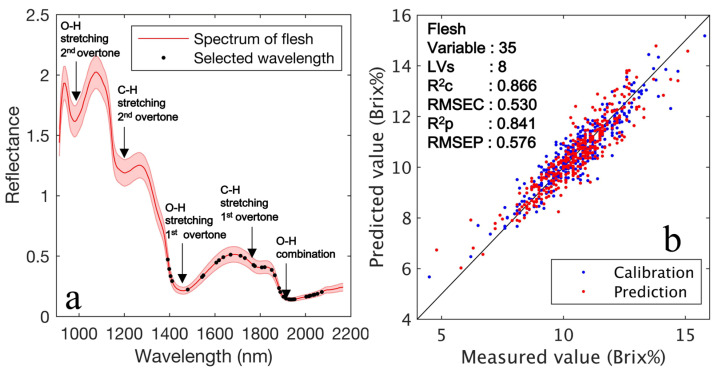
(**a**) Total of 35 key wavelengths (black points) selected by CARS method. (**b**) PLSR calibration result with training data set and prediction with testing dataset using the selected 35 key wavelengths and 8 LVs; the $R^{2}{}_{c}$ and RMSEC are 0.866 and 0.530, whereas $R^{2}{}_{p}$ and RMSEP are 0.841 and 0.576, respectively.

The model constructed from the raw spectra extracted from the fruit ROI had the highest prediction accuracy, with RMSEP and $R^{2}{}_{p}$ values of 0.500 and 0.880, respectively. The accuracy of the models was not much different compared with the fruit and flesh ROIs. Because the ratio of achene pixels was low (8.7%), it had less effect on the model constructed based on the averaged spectrum. Variable selection using CARS led to a lower PLSR factor (LVs). The PLSR model was more stable owing to its fewer latent variables as regression coefficients become noisy with an increase in the number of latent variables. A smaller PLS factor reduces the likelihood of overfitting and makes the model more stable. Especially when considering practical applications, it is better to adopt a model with a low PLS factor to apply to unknown samples of various variations. Thus, the mode masked by gray in Table 1 with variable selection by CARS with the highest $R^{2}{}_{p}$ was applied to the visualization step. Figure 9 shows the wavelength selected by CARS and the relation between the measured and predicted Brix values. Raw spectra extracted from the flesh ROI were employed for the model. Figure 9a depicts the 35 wavelengths (black points) selected using the CARS method. These selected wavelengths are associated with C-H (approximately 1420 and 1780 nm) and O-H (approximately 1900 nm). Figure 9b depicts the relationship between the measured and predicted Brix values obtained by PLSR calibration for the training (blue) and testing dataset (red). Eight PLS factors (LVs) were selected as the optimum number for the PLSR calibration model using 35 critical wavelengths. The PLSR calibration model had substantial prediction accuracy; its $R^{2}{}_{c}$ and RMSEC were 0.866 and 0.530, $R^{2}{}_{p}$, and RMSEP were 0.841 and 0.576, respectively. Because the difference in accuracy between the calibration and prediction datasets was small, the PLSR model did not overfit the data. The prediction accuracy $R^{2}{}_{p}$, and RMSEP of a model proposed in a prior study [25], which visualized the total water-soluble sugar (TWSS) in strawberries with red skin using NIR-HSI (1000–2500 nm), were 0.774 and 6.459 mg∙g^−1^, respectively. Note that TWSS is the total amount of sugar measured using HPLC and is strongly correlated with the Brix value. The PLSR model used in this study exhibited a higher prediction accuracy than prior NIR-HSI investigations. Moreover, the prediction results were equally high compared with the sugar content prediction results of FT-NIR spectrometry ($R^{2}{}_{p}$ and RMSEP were 0.85 and 0.58, respectively) [36].

### 3.3. Visualization of the Sugar Content Distribution

**Figure 10:**
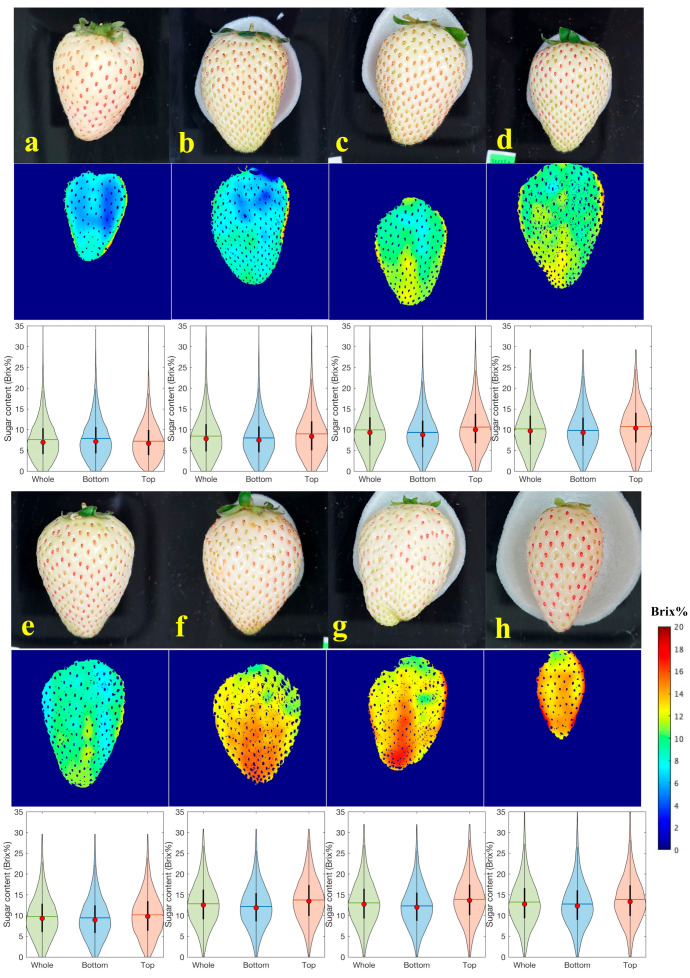
Prediction images and violin plots of Brix values using the PLSR model for white strawberries. Violin plots represent the distribution of pixel Brix values for each ROI (whole flesh, bottom flesh, and top flesh). In order to display representative samples, samples were selected from the lowest to the highest sugar content and arranged in (**a**–**h**) order.

Figure 10 depicts heatmap images of Brix prediction for each flesh ROI using the developed PLSR model and violin plots denoting the distribution of pixel Brix values for the whole fruit, bottom, and top. In order to display representative samples, samples were selected from the lowest to the highest sugar content and arranged in alphabetical order. The color scale indicates the predicted Brix values of the strawberries. Our heatmap and violin plot using the flesh ROI mask remove approximately 8.7% (ratio of achene pixels) of unnecessary pixel information that is not needed for the flesh of fruit surface evaluation. The differences between the Brix values for each strawberry were successfully visualized. In an earlier study [25,26], the characteristics of the flesh parts could not be observed owing to the color of the achene. By contrast, variations in the sugar content of local flesh parts were observed in our heatmap images. Furthermore, violin plots showed the sugar content distribution of the flesh in the whole fruit, bottom, and top. The heatmap images have the benefit of assessing Brix size and distribution. Simultaneously, violin plots helped to statistically determine the differences in Brix between samples and sample parts. Visualizing spatial distribution and violin plots is an excellent way to evaluate strawberries that can be offered to consumers or used as a selection criterion. Because the wavelength (913–2166 nm) of NIR-HSI used in the proposed method does not depend on pigment information, such as anthocyanin, this evaluation method can also be applied to red strawberries.

## 4. Conclusions

In this study, a new method to evaluate the sugar content of white strawberries using NIR-HSI was proposed. A preprocessing method combining PCA and image processing was developed to automatically separate flesh and achene on the fruit surface. The PLSR model constructed from the raw spectra extracted from the flesh ROI exhibited good prediction accuracy with RMSEP and $R^{2}{}_{p}$ of 0.576 and 0.841, respectively, and included a relatively low number of PLS factors. This model demonstrated good prediction performance. The characteristics of the sugar content distribution in the flesh of white strawberries were depicted using the produced Brix heatmap images and violin plots.

These findings suggest that NIR-HSI can be used for noncontact evaluation of the quality of white strawberries. The key advantage of NIR-HSI is its ability to assess fruit without damaging it. If the HSI data of strawberries growing in the field can be measured over time, novel criteria for judging ripeness from visualization of variations in the distribution of Brix values can be developed. Although we focused on Brix as a measure of sugar content in this research, the same approach can be extended to other indicators of quality, such as acidity, hardness, and damage, by increasing the number of objective variables.

The results of this study are expected to have practical applications, particularly for fruit sorting. A sorting system can be constructed using a conveyor belt. NIR-HSI is useful for adding value because it provides detailed information, such as the sweetness of each part of the fruit, in the form of images and violin plots. It can also be used to obtain information on the entire surface of the fruit by inverting and measuring it. Existing optical sorting systems measure only a single representative value and thus provide relatively little information. Spatial, spectral information enables quantitative evaluation of other characteristics, such as blemishes, and fruit sorting systems based on such sensors are expected to provide a powerful basis for quality evaluation in determining strawberry prices.

## Figures and Tables

**Table 1:** Results of PLSR model search. The table is sorted in ascending $R^{2}{}_{p}$.

Pretreat Method	Variable and LVs Selection	ROI	Variable	LVs	RMSECV (Brix%)	RMSEC (Brix%)	RMSEP (Brix%)	*R* ^2^ *_C_*	*R* ^2^ *_P_*
SNV + 2nd derivative	CARS + CV	Achene	49	4	1.095	1.029	1.043	0.494	0.477
2nd derivative	CARS + CV	Achene	75	5	1.068	0.997	1.038	0.525	0.482
Raw	CARS + CV	Achene	100	11	0.906	0.825	0.904	0.674	0.607
SNV	CV	Achene	200	16	0.798	0.630	0.805	0.810	0.688
SNV	CARS + CV	Achene	31	9	0.776	0.727	0.799	0.747	0.693
SNV + 2nd derivative	CV	Achene	185	10	0.841	0.735	0.793	0.742	0.697
2nd derivative	CV	Achene	185	19	0.773	0.614	0.767	0.820	0.717
2nd derivative	CARS + CV	Flesh	145	4	0.739	0.703	0.742	0.764	0.735
2nd derivative	CARS + CV	Fruit	157	4	0.731	0.694	0.728	0.770	0.745
Raw	CV	Achene	200	19	0.722	0.567	0.716	0.846	0.753
SNV	CARS + CV	Flesh	37	8	0.572	0.537	0.714	0.862	0.755
SNV + 2nd derivative	CARS + CV	Fruit	88	4	0.680	0.649	0.692	0.799	0.769
SNV + 2nd derivative	CARS + CV	Flesh	34	5	0.691	0.645	0.683	0.801	0.775
SNV	CARS + CV	Fruit	60	9	0.630	0.579	0.632	0.839	0.808
Raw	CARS + CV	Fruit	26	9	0.600	0.566	0.633	0.847	0.808
Raw	CARS + CV	Flesh	35	8	0.558	0.530	0.576	0.866	0.841
SNV + 2nd derivative	CV	Fruit	185	15	0.506	0.436	0.555	0.909	0.852
2nd derivative	CV	Flesh	185	20	0.537	0.442	0.553	0.907	0.853
2nd derivative	CV	Fruit	185	20	0.516	0.433	0.541	0.910	0.859
SNV	CV	Fruit	200	15	0.533	0.459	0.527	0.899	0.866
SNV	CV	Flesh	200	15	0.573	0.476	0.523	0.891	0.869
Raw	CV	Flesh	200	17	0.528	0.450	0.520	0.903	0.870
SNV + 2nd derivative	CV	Flesh	185	16	0.517	0.438	0.512	0.908	0.874
Raw	CV	Fruit	200	19	0.511	0.413	0.500	0.918	0.880

## Data Availability

The datasets generated and/or analyzed during the current study are available from the corresponding author on reasonable request.
